# Two Old Dogs, One New Trick: A Review of RNA Polymerase and Ribosome Interactions during Transcription-Translation Coupling

**DOI:** 10.3390/ijms20102595

**Published:** 2019-05-27

**Authors:** Adam B. Conn, Stephen Diggs, Timothy K. Tam, Gregor M. Blaha

**Affiliations:** Department of Biochemistry, University of California, Riverside, CA 92521, USA; aconn003@ucr.edu (A.B.C.); sdiggs001@ucr.edu (S.D.); ttam004@ucr.edu (T.K.T.)

**Keywords:** bacteria, transcription, translation, coupling, RNAP, ribosome, ribosomal subunits, RfaH, NusG, nascent RNA

## Abstract

The coupling of transcription and translation is more than mere translation of an mRNA that is still being transcribed. The discovery of physical interactions between RNA polymerase and ribosomes has spurred renewed interest into this long-standing paradigm of bacterial molecular biology. Here, we provide a concise presentation of recent insights gained from super-resolution microscopy, biochemical, and structural work, including cryo-EM studies. Based on the presented data, we put forward a dynamic model for the interaction between RNA polymerase and ribosomes, in which the interactions are repeatedly formed and broken. Furthermore, we propose that long intervening nascent RNA will loop out and away during the forming the interactions between the RNA polymerase and ribosomes. By comparing the effect of the direct interactions between RNA polymerase and ribosomes with those that transcription factors NusG and RfaH mediate, we submit that two distinct modes of coupling exist: Factor-free and factor-mediated coupling. Finally, we provide a possible framework for transcription-translation coupling and elude to some open questions in the field.

## 1. Introduction

In bacterial cells, the lack of a physical barrier allows transcription and translation machineries to mingle, thus enabling concurrent translation of an mRNA while it is being transcribed, in a process known as transcription-translation coupling [1,2]. Due to this coupling, ribosomes translating the nascent RNA trail the transcribing RNA polymerase (RNAP) [3], bringing both physically close to each other [4,5]. This proximity of the transcribing RNAP and the first translating ribosome rationalizes long-standing observations, such as transcription polarity and transcription attenuation.

**Figure 1:**
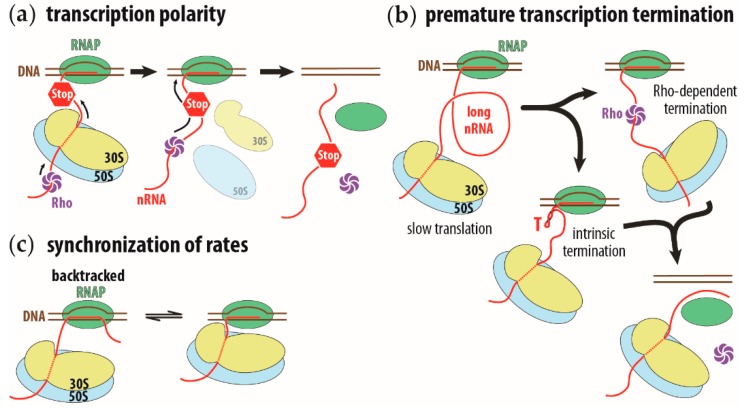
Schematic representation of transcription polarity, premature transcription termination on long intervening nascent RNA, and synchronization of transcription and translation rates. (**a**) Transcription polarity is caused by a premature stop codon (STOP sign) on the nascent RNA (nRNA, in red). Translation of the nascent RNA will terminate and the ribosome (in yellow and blue, for small and large ribosomal subunits, respectively) will prematurely dissociate from the nascent RNA. This allows the Rho transcription termination factor (in purple) to reach the RNA polymerase (RNAP, in green) and induce premature transcription termination. (**b**) A long intervening nascent RNA allows the Rho factor to bind ahead of the ribosome or allow the intrinsic transcription terminator to fold (hairpin structure indicated with a red capital T). In both instances, transcription terminates. (**c**) Synchronization of transcription rate to translation rate. The running ahead of the RNAP will cause the polymerase to pause and backtrack (complex on the left). The translating ribosome will push the RNAP forward and reactivate its transcription activity (complex on the right). This running ahead and pausing to wait for the ribosome synchronizes the transcription rate to the translation rate.

In transcription polarity, a premature stop codon curtails not only the expression of the mutated gene, but also that of all genes on the same polycistronic operon downstream of the mutation. The premature translation termination causes the ribosome to dissociate from the nascent mRNA, allowing the transcription termination factor, Rho, to proceed along the nascent RNA all the way to the RNAP. At the RNAP, the Rho factor induces transcription termination, halting transcription of the downstream genes on the operon [6,7,8] (Figure 1a). Premature transcription termination can occur by simply reducing the rate of translation. The slower speed of the first trailing ribosome will increase the length of the intervening nascent RNA between the ribosome and the RNAP. This longer RNA gap, between the RNAP and the leading ribosome, allows the Rho factor to bind to the nascent RNA, ahead of the ribosome, in direct line to the RNAP [9]. The longer intervening RNA also provides sufficient room for intrinsic terminator signals to fold and cause the termination of transcription [10] (Figure 1b).The intrinsic transcription termination signal consists of a stable hairpin stem loop, followed by a uridine-rich sequence [11].

Programmed decoupling of transcription and translation is also exploited for gene regulation, i.e., transcription attenuation. The most well-known example of transcription attenuation involves the cellular concentration of tryptophan regulating the expression of the *trp* operon. Starvation for tryptophan induces stalling of the first ribosome translating the leader sequence of the operon. The stalled ribosome prevents the formation of a transcription termination signal on the nascent RNA, allowing the RNAP to continue to transcribe downstream genes on the *trp* operon [12,13]. In other instances of transcription attenuation, the stalled ribosome leads to transcription termination, thereby halting the expression of downstream genes [14,15,16].

The proximity between the transcribing RNAP and its trailing ribosome is maintained under different growth conditions [17,18]. A slowing of translation induced by either an antibiotic or a mutation results in a corresponding slowing of transcription [19]. This slowing of translation allows the RNAP to run ahead of the ribosome, where it is more likely to stall and backtrack. While backtracking, the RNAP slides backwards on the nascent RNA and DNA template strand, rezipping the upstream nascent RNA and template DNA while extruding the nascent RNA’s 3’ end out of the NTP entry site. A ribosome trailing closely behind the RNAP will prevent the polymerase from sliding backwards, biasing the polymerase toward the forward direction, extending the nascent RNA [19]. This interplay between the RNAP running ahead and stalling and being reactivated by a trailing ribosome, results in the matching of the RNAP’s speed to that of the ribosome, i.e., the rate of transcription is synchronized to the rate of translation (Figure 1c). Therefore, inhibiting translation leads to a genome-wide stalling of transcription [20]. The stalled RNAP acts as a barrier for the DNA replication machinery, jeopardizing the processivity of replication and, with it, the integrity of the genome [21,22].

Increasing the transcription rate by blocking backtracking also occurs on non-protein encoding genes. Rather than a trailing ribosome, a trailing RNAP suppresses stalling and backtracking of the leading RNAP. The trailing polymerase biasing the leading polymerase toward the forward direction explains the higher overall transcription rate of highly transcribed non-coding operons, such as the rRNA operons (i.e., 85 nts/sec for rRNA vs. 40–55 nts/sec for mRNA) [23,24,25].

## 2. Do Transcription and Translation Occur in the Same Cellular Compartment?

Although transcription and translation are assumed to occur in the same compartment, this assumption has been recently challenged. In *Escherichia coli*, the genome is segregated from the cytoplasm, forming a dense, compact structure in the center of the cell, known as the nucleoid. The nucleoid sequesters nearly all of the cellular RNAPs [26,27] while expelling most of the ribosomes, forcing them to accumulate on the periphery of the nucleoid, particularly at the poles of the cell [27,28,29]. This spatial separation of RNAPs and ribosomes suggests that most of the transcription and translation occurs apart from each other [30].

Tracking of individual ribosomal subunits reveals that most of the translating ribosomes are excluded from the nucleoid, while free ribosomal subunits can enter the nucleoid almost as unhindered as tRNAs and translation factors [30,31,32,33,34]. Similarly, tracking of individual RNAP molecules indicates that free RNAPs can move unhindered within the nucleoid at a rate comparable to that in solution [32]. This unhindered diffusion of the RNAP suggests that the RNAP is, in addition to sliding along individual DNA strands, crossing between DNA strands in its search for a transcription start site [35]. Once a polymerase has found a transcription start site, it initiates and transcribes approximately 100–150 nucleotides before pausing, i.e., promoter proximal pausing [36]. The polymerase pausing presumably enables one or several ribosomes to initiate translation on the nascent RNA and catch up to the polymerase, thus establishing the coupling of transcription and translation.

**Figure 2:**
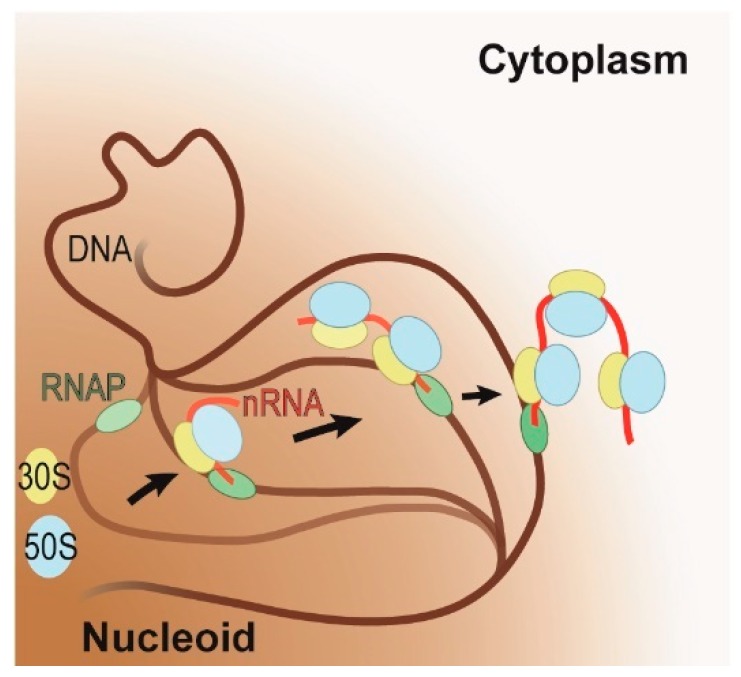
Schematic representation of the coupling of transcription and translation on highly expressed genes under fast growing conditions. RNA polymerase (RNAP, in green) initiates transcription on the DNA (in brown) within the nucleoid (brown shaded area). As soon as the polymerase has transcribed a sufficiently long nascent RNA (in red), translation will ensue (large and small ribosomal subunits in blue and yellow, respectively). During coupling, the active gene is relocalized to the interface of the nucleoid and cytoplasm. The progression of this relocalization is indicated by arrows and by the progressive increase in opacity of the DNA, RNAP, nascent RNA, and ribosomal subunits.

Under fast growing conditions, the transcribing RNAPs cluster on the periphery of the nucleoid, while under slow growing conditions, remain distributed throughout the nucleoid [29,32]. In a nutrient-rich condition, *E. coli* requires fewer genes to satisfy its metabolic needs, therefore allowing it to redirect its resources toward expressing the genes required for fast growth. This results in the aggregation of RNAPs and ribosomes on these few, highly expressed genes. Possibly driven to increase the nucleoid’s conformational flexibility (i.e., entropy) [37], these highly expressed genes move to the periphery of the nucleoid where they cluster (Figure 2) [29,32,38]. This suggests that under fast growing conditions all genes are transcribed at the interface between the nucleoid and ribosome-rich cytoplasm, which further implies that all genes that can support coupling and will have transcription and translation coupled.

Under slow growing conditions, the RNAP remains evenly distributed in the nucleoid [29,32]. Based on the observation that an mRNA undergoes on average 30–60 rounds of translation before it is degraded and only the first round is coupled to transcription [25,39], we estimate that no more than 4% of ribosomes participate in transcription-translation coupling. 10–15% of ribosomes are present as free ribosomal subunits, which can enter the nucleoid nearly unhindered [31]. As the nucleoid occupies nearly half the volume of the cell, it implies that at least 5% of ribosomes are available in the nucleoid. This suggests that, even under slow growing conditions, transcription and translation are coupled. Note that we are implicitly assuming that all protein-encoding genes require or support transcription-translation coupling—an assumption that has not yet been tested.

## 3. Does Transcription-Translation Coupling Result only from the Colocalization of RNAP and Ribosomes on Nascent RNA?

The above described observations of transcription-translation coupling can be explained by the binding of ribosomes to the nascent RNA of a transcribing RNAP; no physical interactions between the transcribing RNAP and the first trailing ribosome must be evoked. This assumption was first challenged by the NMR structure of the complex of ribosomal protein uS10, bound to the transcription factor, NusG [40]. (Ribosomal protein uS10 is also known as ribosomal protein S10 and as transcription factor NusE. Here, we follow the naming convention for ribosomal proteins as set forth in Ban et al. [41].)

NusG stimulates transcription [42,43] as well as translation [44]. Of the two domains of NusG, the N-terminal domain binds to the RNAP, while the C-terminal domain can bind either transcription termination factor Rho [45] or ribosomal protein uS10 [40]. The binding of the N-terminal domain of NusG to the RNAP prevents the polymerase from entering long-lived pauses, thereby increasing the overall transcription rate [46]. On the other hand, the binding of the Rho factor to the C-terminal domain places the transcription termination factor near the nascent RNA. This proximity facilitates the loading of the Rho factor onto the nascent RNA only a short distance away from the RNAP, thereby promoting a Rho-dependent transcription termination. NusG’s ability to prevent prolonged transcription pausing and to recruit the Rho factor to the RNAP explains the apparently contradictory effects NusG exerts on transcription, stimulating both transcription elongation and transcription termination [47,48].

The binding interface of NusG on ribosomal protein uS10 within the NusG:uS10 complex is accessible on the ribosome [40], and residues within this interface are critical for the binding of NusG to the ribosomes both in vitro and in vivo [49]. NusG appears not only to bind the RNAP and the ribosomes on their own, but also to form a physical link between the transcribing RNAP and the trailing ribosome during transcription-translation coupling [40]. Since ribosomal protein uS10 competes with the Rho factor for overlapping sites on NusG’s C-terminal domain [40,50], coupling of the RNAP and the ribosomes via NusG suppresses the recruitment of the Rho factor and, with it, its mediated transcription termination.

Another transcription factor known to physically link the RNAP and the ribosomes is the NusG-paralog RfaH. While NusG associates with the RNAP during expression of almost all genes [36], RfaH regulates the expression of a handful of operons with a specific signal sequence in the 5’ untranslated region (i.e., operon polarity suppressor or *ops* signal) [51]. The coupling brought about by RfaH enables the expression of exogenous, horizontally transferred genes, even if they are not codon-optimized and are missing the translation initiation signals specific for *E. coli* [52].

Like NusG, RfaH consists of two domains. Its N-terminal domain is highly similar to NusG’s and equally reduces transcription pausing. Both proteins even compete for binding to overlapping sites on the RNAP, resulting in a mutually exclusive binding of the factors to the RNAP in vivo [53]. The C-terminal domains of both factors, however, are strikingly different. While the C-terminal domain of NusG adopts an all β-sheet structure and is connected via a flexible linker to its N-terminal domain [54], the C-terminal domain of RfaH folds into an all α-helical structure and intimately interacts with its N-terminal domain [55]. The RNAP will pause upon transcribing the *ops* signal. This allows RfaH to recognize the *ops* sequence on the non-template DNA strand and bind to the polymerase [56,57]. Upon binding of the N-terminal domain, RfaH’s C-terminal domain is released and adopts the all β-sheet structure of NusG [52]. The transformed C-terminal domain then allows RfaH to recruit ribosomal protein uS10 at the same interface that NusG does [53].

Unlike NusG, RfaH does not bind the Rho factor [52]. Therefore, RfaH hampers Rho-dependent transcription termination in two ways. First, it blocks the Rho factor from reaching the RNAP by mediating a tight coupling between the RNAP and the first trailing ribosome. Second, it diminishes NusG’s stimulatory effect on the Rho-dependent termination by competing with NusG for binding to the RNAP.

## 4. Are RNAP and Ribosome only Linked Together by NusG and RfaH or Can They Directly Interact with Each Other?

Early genetic studies uncovered an interaction between RNAP and the small ribosomal subunit [58,59], indicating that mediating factors, such as NusG or RfaH, may not be required for the coupling of transcription and translation. Additionally, several ribosomal proteins also serve as transcription factors on their own. For example, ribosomal protein uS4 inhibits premature termination of ribosomal RNA transcription [60], while ribosomal protein uL2 promotes transcription of genes driven from ribosomal RNA promoters [61]. Ribosomal protein uS10, which binds the C-terminal domain of NusG as discussed above, is an integral part of a transcription antitermination complex and can bind the RNAP on its own [40,62,63,64]. Finally, ribosomal protein bS1 stimulates the recycling of the RNAP during in vitro transcription [65]. Although bS1 binds only weakly to the ribosomes, it is critical for translation initiation [66]. By capturing the mRNA in its unfolded form, bS1 provides the ribosome access to the ribosomal binding site (also known as the Shine–Dalgarno sequence) buried in a local secondary structure [67,68]. This unfolding of the structured mRNA extends not only downstream towards the Shine–Dalgarno region, but also upstream of the bS1 binding site. In some cases, the upstream unfolding is large enough to accommodate a second ribosome, priming the mRNA for a second round of translation [69]. Once translation has ensued, bS1 is unable to dissociate from the ribosome [70]. This suggests that the ribosome-bound bS1 interacts with the RNAP during transcription-translation coupling.

**Figure 3:**
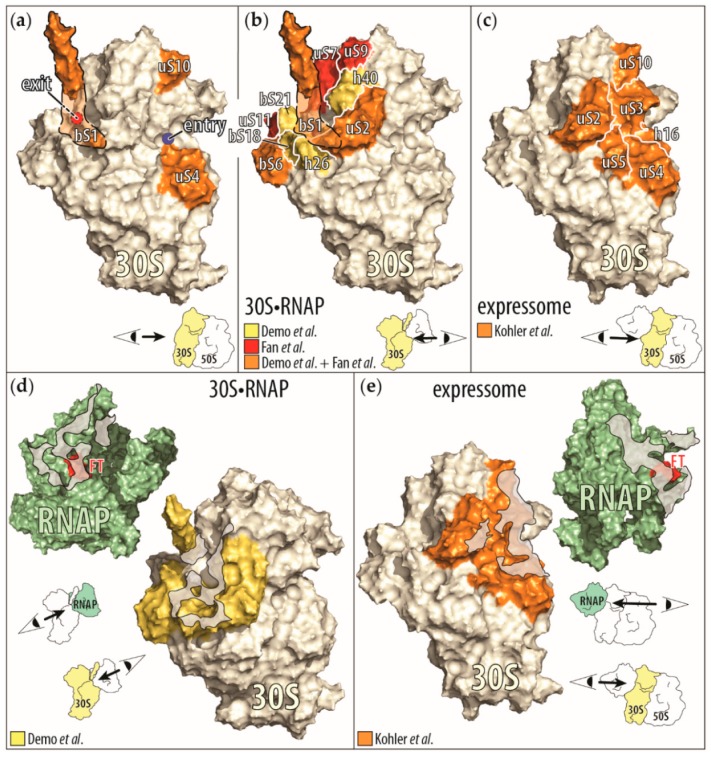
Display of the RNAP-ribosome interactions and contact points identified by biochemical and cryo-EM studies. (**a**) Ribosomal proteins (in orange) that influence the RNAP activity by themselves (bS1 [65], uS4 [60], and uS10 [63]) are mapped onto the small ribosomal subunit (30S), derived from the cryo-EM structure of the small ribosomal subunit bound to the RNAP (30S•RNAP) [71]. Because ribosomal protein bS1 is only partially resolved in this structure, we outlined the approximate position of the remaining protein (orange shaded area). In addition, the mRNA entry (blue circle) and exit (red circle with dashed black border indicating its positions behind bS1) sites on the small ribosomal subunit are indicated. In the right corner is a cartoon representation of the direction of the view displayed of the small ribosomal subunit. (**b**) Ribosomal proteins and RNA helices contacting the RNAP upon binding of the small ribosomal subunit to RNAP. Shown are the proteins identified to be close to the RNAP in the cryo-EM structure of 30S•RNAP in Demo et al. [71] and by chemical crosslinking in Fan et al. [73]. (Proteins observed only in Demo et al. are in yellow, those shared by Demo et al. and Fan et al. are in orange, and those observed only in Fan et al. are in red.) (**c**) Ribosomal proteins (in orange) contacting the RNAP in the cryo-EM structure of a ribosome translating a nascent RNA as it is being synthesized, also known as expressome [72]. Interactions between the C-terminal domain of one of the two α subunits of the RNAP with the ribosome were omitted for clarity. (**d**,**e**) Contact interfaces between the RNAP and the small ribosomal subunit as seen in the cyro-EM structures of the 30S•RNAP complex (**d**) and the expressome (**e**). In both representations, the view is onto the contact areas (gray shaded areas) on the RNAP (green) and on the small ribosomal subunit (yellow). Also indicated is the β flap-tip of the RNAP (red, marked with FT), past which the nascent RNA exits the RNAP to enter the small ribosomal subunit.

Direct interactions between the RNAP and the ribosomes were also observed in the cryo-EM studies of the small ribosomal subunit bound to the RNAP (i.e., the 30S•RNAP complex) and of the ribosome in complex with the RNAP, in which the ribosome is translating the nascent RNA being transcribed by the RNAP (i.e., the expressome) [71,72]. In reconstructions of both complexes, the RNAP binds to the ribosome with its nascent RNA exit site while the RNAP binding sites on the ribosome are distinct and non-overlapping (Figure 3b–e).

In reconstructions of the 30S•RNAP complex, the RNAP is bound close to the 30S subunit site that recognizes the Shine–Dalgarno sequence of mRNAs. The interface between the RNAP and the 30S subunit consists of regions of the β’ and β subunits close to the nascent RNA exit site on the RNAP and of ribosomal proteins uS2, bS6:bS18 heterodimer, bS21, and ribosomal RNA helices 26 and 40. Due to the flexibility of the ribosomal protein bS1, only parts of the protein were visualized in one of the reconstructed 30S•RNAP particles. In this reconstruction, the C-terminal half of ribosomal protein bS1 interacts with the bound RNAP. In our own studies of the RNAP-30S subunit interface, we found, by chemical crosslinking, that the β’ subunit of the RNAP is close to the ribosomal proteins bS1, uS2, bS6, uS7, uS9, and uS11—all proteins surrounding the mRNA exit site of the 30S subunit (Figure 3b,d) [73].

The 30S subunit of all three reconstructed 30S•RNAP particles adopts the same conformation, in which the diameter of the mRNA exit site is widened. This widening of the 30S subunit’s mRNA exit site may allow the nascent RNA to better access the mRNA path on the 30S subunit during translation initiation [71]. Please note that in all 30S•RNAP particles the RNAP is neither transcribing a nascent RNA nor bound to DNA [71]. Therefore, these complexes may not recapitulate a step during translation initiation of the nascent RNA, but simply reflect interactions between 30S subunits and free RNAP that may occur in the nucleoid.

In the reconstruction of the expressome, the RNAP docks to the mRNA entry site of the ribosome, allowing the nascent RNA exiting the RNAP to immediately enter the ribosome. The binding interface on the RNAP consists of regions close to the nascent RNA exit site from all subunits, and on the ribosome it includes ribosomal proteins uS2, uS3, uS4, uS5, and uS10, and helix 16 of the 30S subunit’s ribosomal RNA [72] (Figure 3c,e). In addition, the C-terminal domain of one of the two α subunits of the bound RNAP is bound to the ribosomal protein uS9 and helices 38 and 39 of the 30S subunit’s ribosomal RNA. This RNAP binding site is more than 75 Å distant from that seen in the 30S•RNAP particles [71,72]. Please note that the expressome complex was prepared by translating all of the nascent RNA of a preformed, stable RNAP complex. Furthermore, the physical contact between the RNAP and the ribosome was stabilized by the presence of the chemical cross-linker glutaraldehyde, during the purification of the complex [72,74]. Therefore, the expressome structure may only reflect a complex with minimal nascent RNA between the RNAP and the ribosome [72].

Although the RNAP positions in both structures are distinct, they may be part of a single, overarching cycle of transcription-translation coupling. We propose that similar to the translation initiation of a structured mRNA, during which the 3’ end is repositioned on the 30S subunit from the mRNA exit to the mRNA entry site [75,76], the RNAP is repositioned during translation initiation of the nascent RNA. Therefore, the RNAP-binding site seen in the 30S•RNAP may reflect a state during the beginning phase of the coupling, while the RNAP-binding site seen in the expressome may reflect a state during ongoing coupling along a gene or operon.

In both structures, the NusG- and RfaH-binding sites on the RNAP and the ribosome are too far apart to allow NusG or RfaH to bridge both macromolecules. Therefore, another spatial arrangement between the RNAP and the ribosome must exist that complements already captured and visualized arrangements.

## 5. Does the Length of Intervening RNAs Influence the Interaction between the RNAP and the Ribosome during Coupling?

The structure of the expressome suggests a continuous, static physical connection between the RNAP and the ribosome during coupling. Such a close connection provides a simple explanation for the effects the coupling of translation exerts on transcription. However, the length of the intervening nascent RNA between the RNAP and the ribosome is constantly fluctuating. Because the ribosome steps three nucleotides at a time along the nascent RNA, the ribosome must wait for the RNAP to add three nucleotides, one by one, before taking a step. This causes the length of the intervening RNA to fluctuate between one, two, three, and no extra nucleotides between the RNAP and the ribosome. Such small variations in the length of the nascent RNA may be scrunched in between the RNAP and the ribosome without breaking the interface.

Larger variations are more difficult to reconcile with a static model of the RNAP-ribosome arrangement of the expressome. The transcription and translation rates are dependent on the cellular concentration of different metabolites, i.e., nucleotides and amino acids, respectively. Therefore, these rates will respond differently to concentration fluctuations of these metabolites. These independent responses of transcription and translation will result in varying lengths of intervening nascent RNA. The rate differences are further aggravated by the apparent independent distribution of transcription and translation regulatory elements along genes. (For more specific information on the different regulatory elements for translation, see review by Rodnina [77], and for those for transcription, see review by Artsimovitch [78].) These larger fluctuations in the length of the intervening RNA can be accommodated by a repeated breaking and forming of the expressome, depending on the length of the intervening RNA.

**Figure 4:**
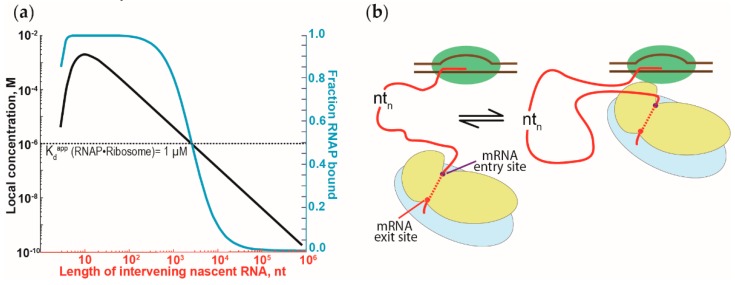
The effect of the tethering of RNAP and ribosomes by nascent RNA on the RNAP•ribosome complex formation. (**a**) Dependence of the RNAP•ribosome complex formation on the length of the intervening nascent RNA. The intervening nascent RNA was modeled as a freely jointed chain. The local concentration of the first trailing ribosome around the RNAP that it is tethered to (left y-axis) and the fraction of the RNAP-ribosome complex formation (in blue, right y-axis) are plotted against the length of the intervening nascent RNA. Local concentration and the fraction of complex formation were calculated, following Conant et al. [79] and Rippe [84]. (**b**) Schematic representation of the binding equilibrium dynamics between the first trailing ribosome (in blue and yellow for large and small ribosomal subunits, respectively) and the RNAP (in green), tethered via the nascent RNA (red). Binding of the RNAP and the ribosome will cause the intervening nascent RNA to loop out and away from the RNAP-ribosome complex.

Because the interactions between RNAP and ribosomes can be repeatedly formed and broken, it suggests that the interactions between both are dynamic. Such a dynamic view of the interactions is supported by the moderate strength of the RNAP affinity for the ribosomes (i.e., a low micromolar dissociation constant of the RNAP•ribosome complex [73]). We suggest that the interactions between the RNAP and ribosomes are not only more dynamic but are possibly independent of the length of the intervening nascent RNA. Due to the tethering via the nascent RNA, the local concentration of the first trailing ribosome close to the RNAP will exceed the dissociation constant of the RNAP•ribosome complex, even with thousands of nucleotides of intervening nascent RNA (Figure 4a) [79]. To accommodate such long intervening RNA, the RNA has to loop out and away from the RNAP-ribosome complex (Figure 4b). Similar looping of the nascent RNA has been attributed to the antitermination observed during transcription of the ribosomal RNA [80,81] and during transcription of the lambda bacteriophage genome [62,79]. Such dynamic binding and dissociation of the RNAP-ribosome complex could explain the stochastic behavior of transcription-translation coupling observed in vivo [82,83]. These lines of argument should also apply to the RNAP-ribosome complex formation mediated by NusG and RfaH.

## 6. What Is the Current Framework for Transcription-Translation Coupling?

We can discern two possible modes of transcription-translation coupling, factor-free and factor-mediated coupling; a distinction also eluded to by others, e.g., [78].

In factor-free coupling, the RNAP is initially recruited to the mRNA exit site of the 30S subunit [71]. During translation initiation, the RNAP relocalizes from the mRNA exit to the mRNA entry site of the 30S subunit [72]. Due to tethering by the nascent RNA and a moderate affinity of the ribosome for the RNAP, the complex between the first trailing ribosome and the RNAP will repeatedly form and dissociate. These frequent encounters between the RNAP and the ribosome enable the coupling to accommodate a fluctuating length of the intervening nascent RNA.

Factor-mediated coupling is most apparent for coupling mediated by the RfaH. Here, the coupling also affects the translation of the nascent RNA, in particular, its initiation [52]. This therefore points to the RfaH already being bound to the RNAP during the first step of translation initiation when the 30S subunit recruits the nascent RNA. Due to RfaH linkage of the RNAP and the trailing ribosome, the spatial arrangement of the RNAP and the ribosome differs from those captured for factor-free coupling.

**Figure 5:**
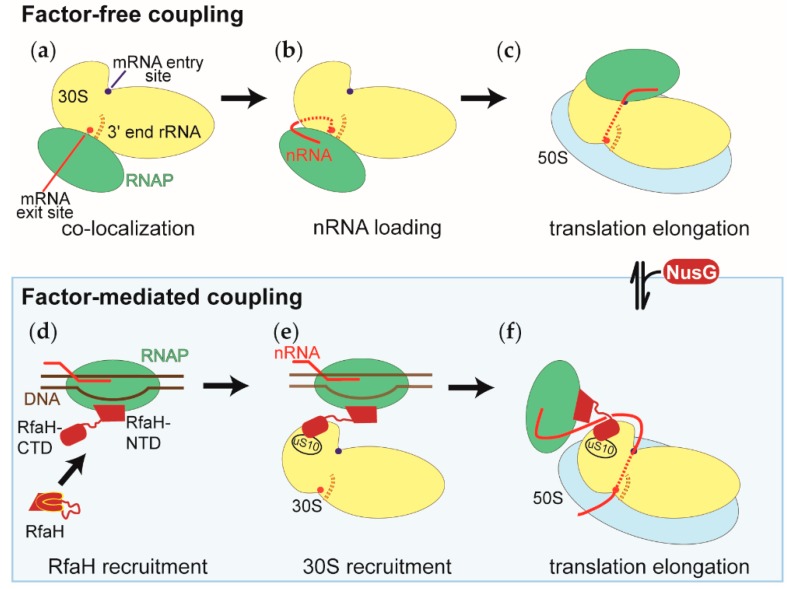
Model of the RNAP-ribosome arrangements during the factor-free and factor-mediated coupling of transcription and translation. The representation of the small ribosomal subunit (30S in yellow) is the same in all panels, with both the RNAP binding sites facing the reader. The RNAP, the large ribosomal subunit (50S), DNA, and the nascent RNA are shown in green, blue, brown, and red, respectively. NusG and RfaH, the factors that physically link the RNAP and the ribosomes during factor-mediated coupling, are shown in dark red. (**a**) Co-localization of the RNAP and the small ribosomal subunits within the nucleoid. (**b**) Recruitment of the nascent RNA to the small ribosomal subunit during the first step of translation initiation. Also shown is the positioning of the 5’ end of the nascent RNA, relative to the 3’ end of the ribosomal RNA of the small ribosomal subunit (3’ end rRNA). In many cases, both ends engage in base pairing interactions. (**c**) During translation initiation, the RNAP relocalizes on the 30S subunit from the mRNA exit site shown in (**a**) and (**b**) to the mRNA entry site. Shown is the RNAP-ribosome complex with the shortest intervening nascent RNA. (**d**) Recruitment of the transcription factor RfaH to the RNAP, which has transcribed and paused at the *ops* signal sequence. The RfaH’s C-terminal domain undergoes a conformational change from an all α helical to an all β sheet structure. (**e**) Recruitment of the small ribosomal subunit (30S) to the RNAP-RfaH complex before initiation of translation. (**f**) During factor-mediated coupling, the RNAP and the ribosome are held close to each other by either the transcription factor RfaH or NusG. The NusG-mediated coupling is established by the binding of NusG to the factor-free coupled RNAP and ribosome.

NusG-mediated coupling appears to be a hybrid of factor-mediated and factor-free coupling. Unlike RfaH, NusG is not recruited at a defined point during the transcription of a gene, but is recruited after the RNAP has cleared a promoter proximal pausing site [36]. This implies that NusG is recruited to the RNAP after translation of the nascent RNA has assisted the polymerase in clearing the pause site. Therefore, NusG can reorganize the RNAP-ribosome arrangement from factor-free to factor-mediated coupling (Figure 5).

## 7. Which Questions Remain?

The discovery of direct physical interactions between the RNAP and the ribosomes hints that individual transcription events can immediately be relayed to the ribosome, affecting its translational activity. Conversely, individual translation events can be relayed to the RNAP, thus affecting its transcriptional activity. The mutual influence of transcription and translation on coupling raises the tantalizing prospective of a novel mechanism of regulation. Any mechanism of the mutual regulation will have to specify: (1) The phases of transcription and translation that are coupled, (2) the functional states of the RNAP and the ribosome that interact, and (3) the effect this regulation exerts on the coupled processes.

Most of our current understanding of transcription-translation coupling was gained from work with a narrow set of model genes and operons under a few conditions. A comprehensive list of genes that support or require coupling for expression remains elusive. Modern genome-wide approaches may overcome this shortcoming in the foreseeable future. It will be interesting to see how this list of genes depends on the presence of NusG or varies with environmental and growth conditions.

Although we focused this review on transcription-translation coupling alone, it is important to realize transcription and translation couples with other critical cellular processes. For example, transcription couples to DNA repair [85,86] and translation couples to protein folding [87,88] and protein translocation [89,90]. The integration of all these coupled processes into a comprehensive view will be required to gain a full appreciation of the effects that transcription-translation coupling exerts on the physiology of the bacterial cell. With the resurgence of interest in transcription-translation coupling, we look forward to new exciting insights into all aspects of coupling and the ramifications for the regulation of gene expression in bacteria.

## Abbreviations

RNAP	RNA polymerase
30S	small ribosomal subunit
50S	large ribosomal subunit
nts	Nucleotides
nts/sec	nucleotides per second
cryo-EM	cryo-Electron Microscopy
